# The RISK pathway and beyond

**DOI:** 10.1007/s00395-017-0662-x

**Published:** 2017-11-15

**Authors:** Xavier Rossello, Derek M. Yellon

**Affiliations:** 1 The Hatter Cardiovascular Institute, University College London, 67 Chenies Mews, London, WC1E 6HX UK; 2 Centro Nacional de Investigaciones Cardiovasculares Carlos III (CNIC), Madrid, Spain

**Keywords:** Cardioprotection, Ischemia/reperfusion injury, Ischemic preconditioning, RISK pathway

## Abstract

Research on cardioprotection has attracted considerable attention during the past 30 years following the discovery of ischemic preconditioning with great advances being made in the field, particularly in the description of the molecular signalling behind this cardioprotective intervention. In a time when basic research is struggling to translate its findings into therapies in the clinical setting, this viewpoint has the intention of presenting to clinical and basic scientists how the reperfusion injury salvage kinase pathway has been described and dissected, as well as highlighting its relevance in cardioprotection.

## The clinical problem in acute myocardial infarction

Time is muscle in patients undergoing acute myocardial infarction (AMI): the less time the coronary artery is occluded, the smaller the infarct size (IS) and the better the outcome for the patient [9, 17]. Although myocardial reperfusion is essential to salvage viable myocardium, it comes at a price in terms of myocardial reperfusion injury which paradoxically also damages the vulnerable post-ischemic myocardium. Studies in animal models of AMI suggest that reperfusion injury may account for a significant contribution to the final myocardial IS [45]. Therefore, targeting myocardial injury using therapies aimed to protect the heart against ischemia/reperfusion injury (IRI), known as cardioprotective therapies [33], remains one of the top ten unmet clinical needs in cardiology [7].

## Ischemic preconditioning: the starting point

Murry et al. published a seminal study demonstrating that several short cycles of non-injurious ischemia and reperfusion significantly protected from a subsequent sustained ischemic insult [29]. This phenomenon, whereby the myocardium can endogenously be protected from lethal IRI, was defined as “ischemic preconditioning” (IPC). This finding, firstly described in dogs, has been subsequently replicated in numerous pre-clinical studies [41], as well as in other organs [42] and in man [43]. The concept of IPC has evolved into “ischemic conditioning”, a broader term that encompasses a number of related endogenous cardioprotective strategies, applied either to the heart (ischemic preconditioning or postconditioning) or from afar (remote ischemic pre-, per- or postconditioning).

Although the translational potential of IPC is inevitably limited by the necessity to apply the intervention before the index ischemia, which is unpredictable in AMI patients, this observation was still of significant importance for two main reasons: (1) infarct size was demonstrated to be potentially modulated through an endogenous mechanism, still considered the most powerful cardioprotective therapy to date, and (2) IPC triggered more than three decades of research [11] in which significant advances have been made in our understanding of the mechanisms underlying IRI and IPC and therefore in the potential development of cardioprotective therapies.

## Origins of the finding of the RISK pathway: necrosis vs apoptosis

During the 90s, the focus of the research on cardioprotection attributed to different types of cellular death resulting from IRI, namely—both apoptosis and necrosis. This was evidenced by terminal deoxynucleotidyl transferase dUTP nick-end labelling and triphenyl tetrazolium chloride staining, respectively [26]. Briefly, necrosis is the form of cell death that occurs following severe cellular damage and includes uncontrolled disruption of organelles, membrane rupture, and does not require adenosine 5′-triphosphate (ATP) [21]. On the other hand, apoptosis is an ATP-dependent programmed cell death that involves cytochrome-c release from injured mitochondria or autocoid cell-surface receptor (Fas Ligand) activation, followed by the downstream propagation of the signal via caspases and other signalling proteins. These in turn cause the formation of nonselective pores in the outer mitochondria or the opening of the mitochondrial permeability transition pore, as well as in DNA cleavage and nuclear degradation [21]. Unlike necrosis, apoptosis does not result in the release of cellular content into the extracellular milieu.

From this early period, pro-apoptotic proteins were the subject of study to try and develop new targets against IRI based upon the hypothesis that it would be possible to salvage cardiomyocytes already committed to die when the signal of programmed cell death is potentially interrupted. It was therefore demonstrated that inhibiting caspases, at the time of reperfusion, limited infarct size in animal models [28]. Besides reducing cell death through the inhibition of pro-apoptotic caspases, the focus was also on using growth factors to antagonize the apoptotic process through the activation of pro-survival proteins—most notably the PI3K and ERK 1/2 pro-survival kinases as a means of protecting ischemic and reperfused myocardium [2, 44].

This leads to the so-called “Reperfusion injury salvage kinase (RISK) pathway” which was first described by Yellon’s group in 2002 whilst assessing the mechanisms underlying the cardioprotective effect induced by urocortin [34]. The use of this growth factor reduced myocardial infarct size and increased the phosphorylation of ERK 1/2 when administered upon reperfusion, these effects being abolished by the co-administration of PD98059 (ERK 1/2 inhibitor) also at reperfusion. The RISK pathway, which is actually a combination of two parallel cascades, PI3K-Akt and MEK1-ERK1/2, was thoroughly dissected through a series of subsequent pharmacological studies where the protective effect of several interventions was blocked with the co-administration of both PI3K and ERK inhibitors at different time-points [14]. In its broadest term, the RISK pathway refers to a group of pro-survival protein kinases, which confer cardioprotection when activated specifically at the time of reperfusion [14, 34].

## Relevance of the RISK pathway

The importance of this pathway is based upon three concepts:

1. The short-term activation of its kinases is protective

The recruitment of pro-survival kinases are protective when *acutely* activated, whilst their chronic activation would be considered to be harmful due to their growth-inducing effect; the chronic activation of the PI3K-Akt cascade is deleterious, as it induces cardiac hypertrophy [30]. Furthermore in the clinical setting, ERK and Akt have been demonstrated to be chronically activated in the failing heart [10]. Importantly, the heart seems to have developed an ability to control phosphorylation of these kinases by the activation of PTEN which has been described as an important “switch” for controlling the growth-induced pathways [27, 31]. To put this in context, acutely activating this survival pathway is ideally suited to the setting of AMI patients who would only require a one-off intervention, at reperfusion, to protect the heart from IRI.

2. This pathway must be activated at the time of early reperfusion for a given cardioprotective therapy to protect against IRI

Following an IPC stimulus, the activation of the RISK pathway was demonstrated to occur at two time-points, following a biphasic pattern response [33, 45]: (1) during the preconditioning cycles, prior to the index ischemic episode, this phase known as the “trigger phase”, and (2) during the onset of reperfusion, known as the early phase of reperfusion. It is believed that the underlying target for protection against reperfusion injury is the mitochondrial permeability transition pore (MPTP) which opens within the first 15 min of reperfusion [12]. Importantly, the link between the RISK pathway and the MPTP has been shown to occur in rat myocytes [5]. Equally relevant is the lack of protection observed when the MPTP is targeted following 15 min of reperfusion with agents that directly affect the pore such as cyclosporin, sanglifehrin and insulin [12, 20]. The importance of the activation of intracellular mediators at the onset of reperfusion was unknown 15 years ago and has substantial clinical implications—this molecular signalling can potentially be mimicked by pharmacological agents to produce benefits for patients undergoing myocardial IRI.

3. The RISK pathway is a universal signalling cascade for cardioprotection

The RISK pathway may be recruited not only by ischemic conditioning, but also by other pharmacological agents such insulin, bradykinin adenosine or statins [33, 45]. It is therefore considered a universal signalling cascade, or a common pathway, shared by most cardioprotective therapies [15].

## RISK and other important pro-survival pathways

Most of the experimental evidence involving the RISK pathway have been carried out in small rodent models of AMI, whereas its central role in large animals is less well-established [8, 36]. Whilst the link between IPC and RISK activation has been demonstrated in human atrial trabeculae [35], its role in remote conditioning is still at an early phase in both large animal models [13, 37] and humans [18].

In addition to the RISK pathway, other signalling cascades have been suggested to mediate the IPC-induced protective effect [16]: the Survivor Activating Factor Enhancement (SAFE) and the NO/PKG pathway. Lecour et al. demonstrated that the administration of TNF-α before index ischemia (used as pharmacologic IPC-mimetic) was cardioprotective without involving the RISK signalling cascade [25]. Four years later, they described that the administration of TNF-α at reperfusion was recruiting an alternative pathway, coined as the SAFE pathway [22–24], and they also linked the activation of this pathway with preconditioning [39]. In humans, it seems that STAT5, instead of STAT3, may play a relevant role in cardioprotection [6, 18]. A third signalling cascade based on the protein kinase G (PKG) and involving nitric oxide has been also proposed to mediate cardioprotection [4].

**Fig. 1:**
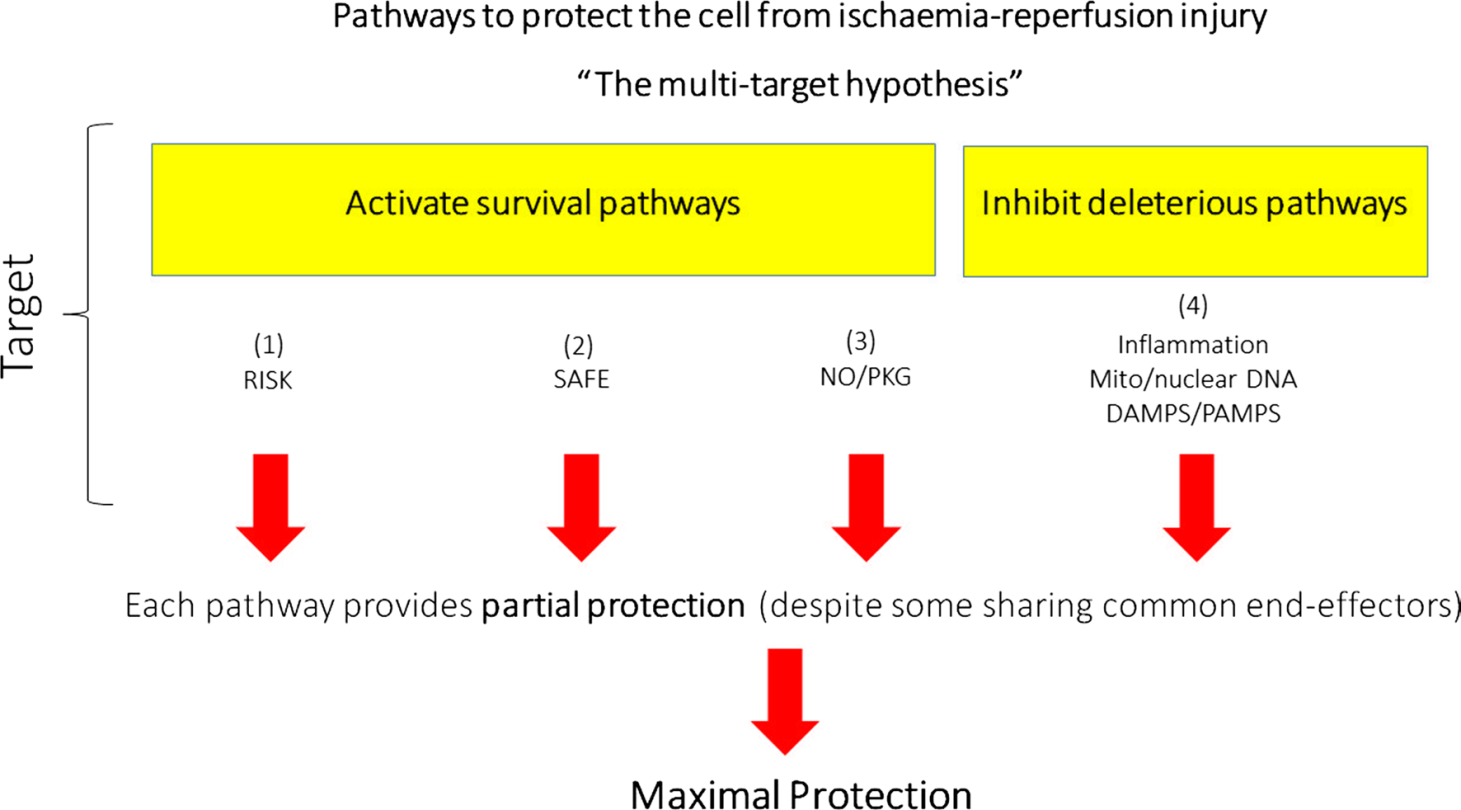
The multi-target hypothesis. *DAMP* danger-associated molecular patterns, *PAMP* pathogen-associated molecular patterns, *RISK* reperfusion injury salvage kinase, *SAFE* Survivor Activating Factor Enhancement

The ability to manipulate and up-regulate pro-survival kinase cascades during the early reperfusion phase provides a potential approach to limiting IRI-induced cell death. Indeed, the use of pharmacological agents targeting such pathways is a feasible intervention which can be applied at the onset of myocardial reperfusion for patients presenting with an ongoing AMI*, either in the ambulance or the cath lab. Therefore, s*trategies enhancing these pro-survival pathways are an attractive target to develop adjuvant therapies to be used alongside cardiac catheterization. The synergistic activation of several pathways using combination therapies represents an attractive target in this context. In the experimental setting, matrix metalloproteinase inhibition has been demonstrated to be protective against IRI through an MPTP-independent pathway, and more importantly, to provide an additive effect to the protection observed following inhibition of MPTP opening [3]. Based on the assumption that insulin and exenatide activate cardioprotective pathways different from those of remote ischemic conditioning (RIC), both therapies have also demonstrated to provide additive effects with RIC on infarct size reduction in pigs [1]. Others have demonstrated that targeting two specific isoforms of PKC (a combined treatment with an ε-PKC activator before ischaemia and δ-PKC inhibitor at the onset of reperfusion) has been proved to induce greater protection against IRI than the treatment with each peptide alone [19]. Moreover, we believe that the RISK pathway as well as the other survival pathways (and their combinations) are by no means the singular route to cardioprotection. To obtain maximum protection, there is still a need to assess complementary and parallel pro-survival mechanisms that can potentially be targeted simultaneously—i.e. mitochondrial and Genomic DNA and the activation of the inflammasome complex, which would target other types of cell death including pyroptosis and necroptosis, both of which are forms of inflammatory cell death [38, 40]. The figure illustrates this “multi-target hypothesis” (Fig. 1).

As such, future research should focus not only on improving the potency of the RISK pathway kinases (i.e. assessing the impact of the relevant isoforms for each kinase [32]) but also defining alternative pathways and new mechanisms to study the synergistic effect of combination therapies which gives the cell its best possible chance of survival.

## Abbreviations

AMI: Acute myocardial infarction
IPC: Ischemic preconditioning
IRI: Ischemia/reperfusion injury
IS: Infarct size
MPTP: Mitochondrial permeability transition pore
RIC: Remote ischemic conditioning
RISK: Reperfusion injury salvage kinase
SAFE: Survivor Activating Factor Enhancement

## References

[1] Alburquerque-Béjar JJ, Barba I, Inserte J, Miró-Casas E, Ruiz-Meana M, Poncelas M, Vilardosa Ú, Valls-Lacalle L, Rodríguez-Sinovas A, Garcia-Dorado D (2015). Combination therapy with remote ischaemic conditioning and insulin or exenatide enhances infarct size limitation in pigs. Cardiovasc Res.

[2] Baxter GF, Mocanu MM, Brar BK, Latchman DS, Yellon DM (2001). Cardioprotective effects of transforming growth factor-beta1 during early reoxygenation or reperfusion are mediated by p42/p44 MAPK. J Cardiovasc Pharmacol.

[3] Bell RM, Kunuthur SP, Hendry C, Bruce-Hickman D, Davidson S, Yellon DM (2013). Matrix metalloproteinase inhibition protects CyPD knockout mice independently of RISK/mPTP signalling: a parallel pathway to protection. Basic Res Cardiol.

[4] Cohen MV, Downey JM (2007). Cardioprotection: spotlight on PKG. Br J Pharmacol.

[5] Davidson SM, Hausenloy D, Duchen MR, Yellon DM (2006). Signalling via the reperfusion injury signalling kinase (RISK) pathway links closure of the mitochondrial permeability transition pore to cardioprotection. Int J Biochem Cell Biol.

[6] Davidson SM, Yellon DM (2012). STAT5 fits the RISK profile for cardioprotection. JAK-STAT.

[7] Fuster V (2014). Top 10 cardiovascular therapies and interventions for the next decade. Nat Rev Cardiol.

[8] Gent S, Skyschally A, Kleinbongard P, Heusch G (2017). Ischemic preconditioning in pigs: a causal role for signal transducer and activator of transcription 3. Am J Physiol Heart Circ Physiol.

[9] Gersh BJ, Stone GW, White HD, Holmes DR (2005). Pharmacological facilitation of primary percutaneous coronary intervention for acute myocardial infarction: is the slope of the curve the shape of the future?. JAMA.

[10] Haq S, Choukroun G, Lim H, Tymitz KM, Del-Monte F, Gwathmey J, Grazette L, Michael A, Hajjar R, Force T, Molkentin JD (2001). Differential activation of signal transduction pathways in human hearts with hypertrophy versus advanced heart failure. Circulation.

[11] Hausenloy DJ, Barrabes JA, Botker HE, Davidson SM, Di Lisa F, Downey J, Engstrom T, Ferdinandy P, Carbrera-Fuentes HA, Heusch G, Ibanez B, Iliodromitis EK, Inserte J, Jennings R, Kalia N, Kharbanda R, Lecour S, Marber M, Miura T, Ovize M, Perez-Pinzon MA, Piper HM, Przyklenk K, Schmidt MR, Redington A, Ruiz-Meana M, Vilahur G, Vinten-Johansen J, Yellon DM, Garcia-Dorado D (2016). Ischaemic conditioning and targeting reperfusion injury: a 30 year voyage of discovery. Basic Res Cardiol.

[12] Hausenloy DJ, Duchen MR, Yellon DM (2003). Inhibiting mitochondrial permeability transition pore opening at reperfusion protects against ischaemia–reperfusion injury. Cardiovasc Res.

[13] Hausenloy DJ, Iliodromitis EK, Andreadou I, Papalois A, Gritsopoulos G, Anastasiou-Nana M, Kremastinos DT, Yellon DM (2012). Investigating the signal transduction pathways underlying remote ischemic conditioning in the porcine heart. Cardiovasc Drugs Ther.

[14] Hausenloy DJ, Tsang A, Mocanu MM, Yellon DM (2005). Ischemic preconditioning protects by activating prosurvival kinases at reperfusion. Am J Physiol Heart Circ Physiol.

[15] Hausenloy DJ, Tsang A, Yellon DM (2005). The reperfusion injury salvage kinase pathway: a common target for both ischemic preconditioning and postconditioning. Trends Cardiovasc Med.

[16] Heusch G (2015). Molecular basis of cardioprotection: signal transduction in ischemic pre-, post-, and remote conditioning. Circ Res.

[17] Heusch G, Gersh BJ (2017). The pathophysiology of acute myocardial infarction and strategies of protection beyond reperfusion: a continual challenge. Eur Heart J.

[18] Heusch G, Musiolik J, Kottenberg E, Peters J, Jakob H, Thielmann M (2012). STAT5 activation and cardioprotection by remote ischemic preconditioning in humans novelty and significance. Circ Res.

[19] Inagaki K (2003). Additive protection of the ischemic heart ex vivo by combined treatment with -protein kinase C inhibitor and -protein kinase C activator. Circulation.

[20] Jonassen AK, Sack MN, Mjøs OD, Yellon DM (2001). Myocardial protection by insulin at reperfusion requires early administration and is mediated via Akt and p70s6 kinase cell-survival signaling. Circ Res.

[21] Konstantinidis K, Whelan RS, Kitsis RN (2012). Mechanisms of cell death in heart disease. Arterioscler Thromb Vasc Biol.

[22] Lacerda L, Somers S, Opie LH, Lecour S (2009). Ischaemic postconditioning protects against reperfusion injury via the SAFE pathway. Cardiovasc Res.

[23] Lecour S (2009). Multiple protective pathways against reperfusion injury: a SAFE path without Aktion?. J Mol Cell Cardiol.

[24] Lecour S (2009). Activation of the protective Survivor Activating Factor Enhancement (SAFE) pathway against reperfusion injury: does it go beyond the RISK pathway?. J Mol Cell Cardiol.

[25] Lecour S, Suleman N, Deuchar GA, Somers S, Lacerda L, Huisamen B, Opie LH (2005). Pharmacological preconditioning with tumor necrosis factor-alpha activates signal transducer and activator of transcription-3 at reperfusion without involving classic prosurvival kinases (Akt and extracellular signal-regulated kinase). Circulation.

[26] McCully JD, Wakiyama H, Hsieh Y-J, Jones M, Levitsky S (2004). Differential contribution of necrosis and apoptosis in myocardial ischemia–reperfusion injury. Am J Physiol Heart Circ Physiol.

[27] Mensah K, Mocanu MM, Yellon DM (2005). Failure to protect the myocardium against ischemia/reperfusion injury after chronic atorvastatin treatment is recaptured by acute atorvastatin treatment: a potential role for phosphatase and tensin homolog deleted on chromosome ten?. J Am Coll Cardiol.

[28] Mocanu MM, Baxter GF, Yellon DM (2000). Caspase inhibition and limitation of myocardial infarct size: protection against lethal reperfusion injury. Br J Pharmacol.

[29] Murry CE, Jennings RB, Reimer KA (1986). Preconditioning with ischemia: a delay of lethal cell injury in ischemic myocardium. Circulation.

[30] Nagoshi T, Matsui T, Aoyama T, Leri A, Anversa P, Li L, Ogawa W, Del-Monte F, Gwathmey JK, Grazette L, Hemmings BA, Hemmings B, Kass DA, Champion HC, Rosenzweig A (2005). PI3K rescues the detrimental effects of chronic Akt activation in the heart during ischemia/reperfusion injury. J Clin Invest.

[31] Rossello X, Riquelme JA, Davidson SM, Yellon DM (2017). Role of PI3K in myocardial ischaemic preconditioning: mapping pro-survival cascades at the trigger phase and at reperfusion. J Cell Mol Med.

[32] Rossello X, Riquelme JA, He Z, Taferner S, Vanhaesebroeck B, Davidson SM, Yellon DM (2017). The role of PI3Kα isoform in cardioprotection. Basic Res Cardiol.

[33] Rossello X, Yellon DM (2016). A critical review on the translational journey of cardioprotective therapies!. Int J Cardiol.

[34] Schulman D, Latchman DS, Yellon DM (2002). Urocortin protects the heart from reperfusion injury via upregulation of p42/p44 MAPK signaling pathway. Am J Physiol Heart Circ Physiol.

[35] Sivaraman V, Hausenloy DJ, Wynne AM, Yellon DM (2010). Preconditioning the diabetic human myocardium. J Cell Mol Med.

[36] Skyschally A, van Caster P, Boengler K, Gres P, Musiolik J, Schilawa D, Schulz R, Heusch G (2009). Ischemic postconditioning in pigs: no causal role for RISK activation. Circ Res.

[37] Skyschally A, Gent S, Amanakis G, Schulte C, Kleinbongard P, Heusch G (2015). Across-species transfer of protection by remote ischemic preconditioning with species-specific myocardial signal transduction by reperfusion injury salvage kinase and survival activating factor enhancement pathways novelty and significance. Circ Res.

[38] Smith CCT, Yellon DM (2011). Necroptosis, necrostatins and tissue injury. J Cell Mol Med.

[39] Suleman N, Somers S, Smith R, Opie LH, Lecour SC (2008). Dual activation of STAT-3 and Akt is required during the trigger phase of ischaemic preconditioning. Cardiovasc Res.

[40] Takahashi M (2011). Role of the inflammasome in myocardial infarction. Trends Cardiovasc Med.

[41] Wever KE, Hooijmans CR, Riksen NP, Sterenborg TB, Sena ES, Ritskes-Hoitinga M, Warlé MC (2015). Determinants of the efficacy of cardiac ischemic preconditioning: a systematic review and meta-analysis of animal studies. PLoS ONE.

[42] Wever KE, Menting TP, Rovers M, van der Vliet JA, Rongen GA, Masereeuw R, Ritskes-Hoitinga M, Hooijmans CR, Warlé M (2012). Ischemic preconditioning in the animal kidney, a systematic review and meta-analysis. PLoS ONE.

[43] Yellon DM, Alkhulaifi AM, Pugsley WB (1993). Preconditioning the human myocardium. Lancet.

[44] Yellon DM, Baxter GF (1999). Reperfusion injury revisited: is there a role for growth factor signaling in limiting lethal reperfusion injury?. Trends Cardiovasc Med.

[45] Yellon DM, Hausenloy DJ (2007). Myocardial reperfusion injury. N Engl J Med.

